# Stem torsion in total hip replacement: CT measurements in 60 patients

**DOI:** 10.3109/17453674.2010.524596

**Published:** 2010-10-08

**Authors:** Ernst Sendtner, Schuster Tibor, Roman Winkler, Michael Wörner, Joachim Grifka, Tobias Renkawitz

**Affiliations:** ^1^Department of Orthopedic Surgery, Regensburg University Medical Center, Regensburg; ^2^Institute of Medical Statistics and Epidemiology, Munich Technical University, Munich, Germany

## Abstract

**Background and purpose:**

The clinical results of THR may be improved by correct femoral torsion. We evaluated the stem position by postoperative CT examination in 60 patients.

**Methods:**

60 patients requiring total hip arthroplasty were prospectively enrolled in this study. Minimally invasive THR was performed (anterior approach) in a lateral decubitus position and each patient underwent a postoperative CT examination. The position of the stem was evaluated by an independent external institution.

**Results:**

Stem torsion ranged from –19° retrotorsion to 33° antetorsion. Normal antetorsion (i.e 10–15° according to Tönnis) was present in 5 of 60 patients, so the prevalence of abnormal stem antetorsion was 92% (95% CI: 82–97). We found a stem antetorsion outside the range of 0–25° in 21 of 60 hips. Women had a higher mean stem antetorsion (8.0° (SD 11)) than men (1.5° (SD 10)).

**Interpretation:**

Postoperative stem antetorsion shows a high variability and is gender-related. We suggest precise assessment of stem antetorsion intraoperatively by means of computer navigation, preparing the femur first. In abnormal stem antetorsion, the cup position can be adjusted using a combined anteversion concept; alternatively, modular femoral components or stems with retroverted or anteverted necks (“retrostem”) could be used.

Femoral antetorsion, considered to be normal within 10–15° (Tönnis and Heinecke 1999), may influence the function of total hip replacement. A consistent method of measuring femoral antetorsion was described by Murphy et al. (1987). Since then, there have been reports on femoral antetorsion measured by means of anatomical investigations or CT scans with or without 3D reconstruction, but relatively little has been reported about stem antetorsion. We investigated stem antetorsion after total hip replacement by use of CT scans and 3D reconstructions. We hypothesized that postoperative stem antetorsion shows a high variability as it is reported for the native femoral antetorsion. In addition to presenting our results, we discuss the implications of stem antetorsion for work flow techniques and implant design in total hip replacement.

## Patients and methods

This study was conducted after obtaining authorization by the institutional ethical board (no. 06/100) and the Federal Office for Radiation Protection (Z5-22462/2-2007-008). Between September 2007 and October 2008, 60 patients requiring total hip arthroplasty for primary osteoarthritis were prospectively enrolled in this single-center study. Exclusion criteria were arthritis secondary to hip dysplasia, posttraumatic deformities of the pelvis, and age below 50 years at the time of surgery. After having obtained written consent from each patient regarding participation, we inserted the uncemented stem using minimally invasive hip arthroplasty. Each patient underwent a pelvic CT examination 3–5 weeks after surgery. The mean age of the 60 patients (38 women) was 68 (50–81) years.

### Surgery

All operations were done by 2 senior consultants through a direct anterior approach with the patient in a lateral decubitus position (Michel and Witschger 2007). We used press-fit cup components (Pinnacle; DePuy, Warsaw, IN) and cement-free hydroxyapatite-coated straight (0° antetorsion) stems (Corail; DePuy). The stem was inserted in the most stable position possible provided by the individual anatomy; no attempt was made to achieve a particular stem antetorsion.

### Measurement of antetorsion

**Figure 1.**
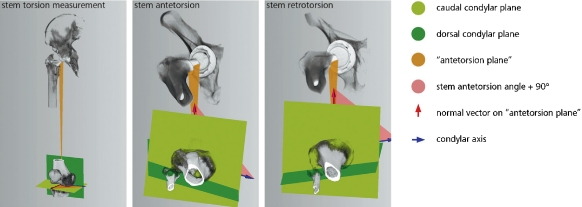
Stem antetorsion measurement. The mechanical axis was defined by 2 points: the center of the femoral head and the center of the caudal contact points of the femoral condyles. “Antetorsion plane” was defined by a third point on the prosthesis representing the direction of the neck. A caudal condylar plane was created orthogonal to the mechanical axis. The angle between the condylar axis projected on this plane and the normal to the “antetorsion plane” subtracted by 90° gave the torsion angle.

The position of the stem component on the CT scan was evaluated by an independent external institution (MeVisLab; MeVis, Bremen, Germany). The position of the femoral component was measured 3 times by 3 independent examiners on a 3D reconstruction of the pelvis with image-processing software, using the following method (Figure 1). The stem antetorsion is aligned within the mechanical axis of the femur and dorsal plane of the femoral condyles. The measurment is done without defining the anatomical axis. First, a caudal and dorsal plane of the femoral condyles is defined to calculate the condylar axis. After that, the mechanical axis of the femur is defined by 2 points: the center of the caudal contact points of the femoral condyles and the center of the femoral head. Finally, a third reference point on the prosthesis is defined so that the vector created, originating from this point pointing towards the center of the femoral head, represents the neck of the prosthesis. Now, the normal vector of the plane created from this reference point and both points of the mechanical axis is calculated. The resulting normal vector and the condylar axis are projected onto a plane that is orthogonal to the mechanical axis. We calculated the angle between these vectors and subtracted 90º. The result is the femoral stem antetorsion.

### Statistics

We used the Kolmogorov-Smirnov test for assessment of normal distribution before further statistical analysis. Antetorsion data were descriptively analyzed, reporting means, standard deviation, and minimum and maximum values. We compared the antetorsion data between men and women using unpaired t-tests. The significance level was set at p = 0.05. We used Microsoft Excel and its statistical software, and SigmaStat for Windows version 3.5.

## Results

### Reliability of the CT-based control method

Stem antetorsion measurements using image processing software showed high reliability: the intraclass correlation coefficient (ICC) for 3 measurements of 1 observer was 0.96 (within-observer) and it was 0.95 between 3 observers.

### Stem antetorsion in 60 THRs

**Figure 2.**
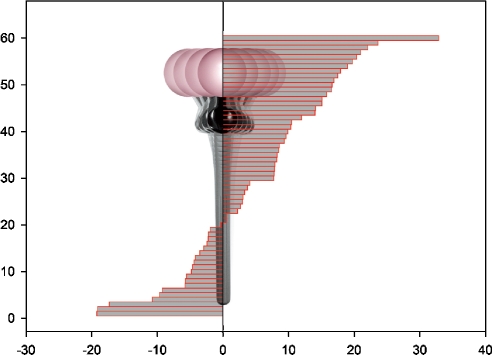
Range of stem torsion in 60 hips.

**Figure 3.**
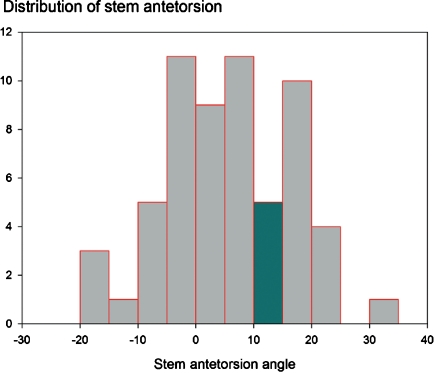
Distribution of stem torsion in 60 hips. The 10–15° of antetorsion shown in green is considered to be the normal stem antetorsion.

The mean stem antetorsion was 5.5° (SD 11). Stem torsion measurements ranged from –19° retrotorsion to 33° antetorsion (Figures 2 and 3). The data match the pattern expected for a normal distribution (p = 0.12; Kolmogorov-Smirnov distance = 0.9). Normal antetorsion (i.e 10–15° according to Tönnis) was present in 5 of 60 hips, so the prevalence of abnormal stem antetorsion was 92% (95% CI: 82–97). We found a stem antetorsion outside the range of 0–25° (i.e. normal antetorsion ± 10°) in 21 of 60 hips. The mean stem antetorsion was higher in women (8.0° (SD 11)) than in men (1.5° (SD 10)) (p = 0.03).

## Discussion

The wide range of stem antetorsion after total hip replacement that we found, as measured by CT scans and 3D reconstructions, correspond well to reports by Wines and McNichol (2006) and Dorr et al. (2009). The differences in the reported means can well be explained by different measurement methods (Wines and McNichol: measurement of stem antetorsion from 2 single CT scans, and no 3D reconstruction; Dorr et al.: referencing to femoral epicondyles, not to the dorsal condylar plane). Our results are valid only for a cementless stem and for a specific implant. We used a minimally invasive surgical procedure; there might be a different stem antetorsion with a traditional or dorsolateral approach, and also between different surgeons. There is also a wide range of native femoral antetorsion (Table). The reported means of antetorsion differ depending on the method used and whether normal or pathologic objects have been used. In cases of osteoarthritis due to developmental dysplasia of the hip, even greater correction angles (–71° up to 35°) for stem antetorsion (custom-made cementless stems) have been reported (Flecher et al. 2006), while the mean preoperative femoral antetorsion was 38.6° (2–86°) (Flecher et al. 2007).

**Table:** Femoral antetorsion measurements

Author	Patients	Range	Mean (SD)	Method	Gender
Maruyama et al. (2001)	100 pelvises, normal joints	–15° to 34°	10° (9)	craniometer, osteometric board	no significant difference in antetorsion
Anda et al. (1991)	33 dysplastic hips, CE < 20°	2° to 45°	20° (12)	2D CT scans	
Husmann et al. (1997)	310 hips with osteoarthritis	0° to 45°	25° (9)	2D CT scans	
Sugano et al. (1998)	30 patients without deformity	3° to 50°	20° (9)	3D reconstructed CT scans	M: 17° ± 7 W: 23° ± 11 but not significant
Sariali et al. (2009)	223 patients with osteoarthritis	0° to 50°	22° (9)	3D reconstructed CT scans	

We found less antetorsion in men. Maruyama et al. (2001) found differences between the sexes for acetabular anteversion angle, anterolateral bowing of the femur, and neck shaft angle, but not for femoral antetorsion (Table). Stem antetorsion is defined by referencing to the mechanical axis of the femur, but the stem position is determined by the anatomical axis following the anterolateral bowing of the femur. Due to this fact, stem antetorsion shows a gender-related difference according to the anterolateral bowing of the femur, and femoral antetorsion does not. Stem antetorsion is also influenced by the femoral helitorsion at 20 mm above the tip of the lesser trochanter, which is correlated to femoral neck antetorsion (r = 0.66), but helitorsion does not necessarily correspond to antetorsion for each particular femur (antetorsion-helitorsion difference over 10° for 25% of the femurs studied) (Husmann et al. 1997). The torsion of the stem is furthermore controlled by the width of the medullary canal and the thickness of the posterior cortex. Thus, stem antetorsion increases additionally if osteoporotic bone is present (Dorr et al. 2009). We found the mean stem antetorsion to be 6.5° greater in females than in males. Even the acetabular anteversion and the combined anteversion has been reported to be greater in females.

The combined anteversion was supposed by Ranawat and Maynard (1991) to be 10–15° higher in females. The mean acetabular anteversion was significantly greater in female pelvises and in 3D reconstructed CT scans of healthy female hips (Maruyama et al. 2001, Vandenbussche et al. 2008): 2.8° and 3.7°, respectively.

Attempts have been made to predict stem antetorsion by computerized 3D preoperative planning (Sariali et al. 2009). Even using an anatomically shaped cementless stem, these authors found that antetorsion of the femoral component was significant different from the anatomical femoral antetorsion. If the stem was implanted manually, the difference between pre- and postoperative antetorsion angles averaged 11° (Jerosch et al. 1998).

Together with the gender-related difference found in our study, these findings suggest that the stem antetorsion originates during stem insertion and that it may be different from preoperative femoral antetorsion. In this regard, our study is limited because we did not do CT preoperatively.

Stem antetorsion could then be estimated intraoperatively by the surgeon, but the intraoperative estimation of femoral antetorsion (and acetabular version) in a total hip arthroplasty is of limited accuracy (Wines and McNicol 2006). The only method by which one could obtain precise information about stem antetorsion would be computer-assisted navigation (Dorr et al. 2009).

In order to achieve good motion of a total hip replacement we suggest intraoperative measurement of stem antetorsion, preparation of the femur first, adjustment of cup orientation to stem antetorsion using the concept of combined anteversion, and/or adjustment of stem antetorsion using modular femoral components.

As an option, stems with retroverted or anteverted necks (“retrostems”) could be manufactured. According to our findings, a stem with a 5–10° correction angle (the difference between the mean stem antetorsion (5.5°) and normal antetorsion (10–15°)) would be feasible. With this correction option, only 3 of 60 hips would have a stem antetorsion outside the range of 0–25°.

## References

[CIT0001] Anda S, Terjesen T, Kvistad KA, Svenningsen S (1991). Acetabular angles and femoral anteversion in dysplastic hips in adults: CT investigation. J Comput Assist Tomogr.

[CIT0002] Dorr LD, Malik A, Dastane M, Wan Z (2009). Combined anteversion technique for total hip arthroplasty. Clin Orthop.

[CIT0003] Flecher X, Argenson JN, Parratte S, Ryembault E, Aubaniac JM (2006). Custom cementless stem for osteoarthritis following developmental hip dysplasia. Rev Chir Orthop Reparatrice Appar Mot.

[CIT0004] Flecher X, Parratte S, Aubaniac JM, Argenson JN (2007). Three-dimensional custom-designed cementless femoral stem for osteoarthritis secondary to congenital dislocation of the hip. J Bone Joint Surg (Br).

[CIT0005] Husmann O, Rubin PJ, Leyvraz PF, de Roguin B, Argenson JN (1997). Three-dimensional morphology of the proximal femur. J Arthroplasty.

[CIT0006] Jerosch J, von Hasselbach C, Filler T, Peuker E, Rahgozar M, Lahmer A (1998). Increasing the quality of preoperative planning and intraoperative application of computer-assisted systems and surgical robots--an experimental study. Chirurg.

[CIT0007] Maruyama M, Feinberg JR, Capello WN, D'Antonio JA (2001). The Frank Stinchfield Award: Morphologic features of the acetabulum and femur: anteversion angle and implant positioning. Clin Orthop.

[CIT0008] Michel MC, Witschger P (2007). MicroHip: a minimally invasive procedure for total hip replacement surgery using a modified Smith-Peterson approach. Ortop Traumatol Rehabil.

[CIT0009] Murphy SB, Simon SR, Kijewski PK, Wilkinson RH, Griscom NT (1987). Femoral anteversion. J Bone Joint Surg (Am).

[CIT0010] Ranawat CS, Maynard MJ (1991). Modern techniques of cemented total hip arthroplasty. Tech Orthopedics.

[CIT0011] Sariali E, Mouttet A, Pasquier G, Durante E, Catone Y (2009). Accuracy of reconstruction of the hip using computerised three-dimensional pre-operative planning and a cementless modular neck. J Bone Joint Surg (Br).

[CIT0012] Sugano N, Noble PC, Kamaric E (1998). A comparison of alternative methods of measuring femoral anteversion. J Comput Assist Tomogr.

[CIT0013] Tönnis D, Heinecke A (1999). Acetabular and femoral anteversion: relationship with osteoarthritis of the hip. J Bone Joint Surg (Am).

[CIT0014] Vandenbussche E, Saffarini M, Taillieu F, Mutschler C (2008). The asymmetric profile of the acetabulum. Clin Orthop.

[CIT0015] Wines AP, McNicol D (2006). Computed tomography measurement of the accuracy of component version in total hip arthroplasty. J Arthroplasty.

